# The World’s Columbian Exposition

**Published:** 1892-03

**Authors:** 


### EDITORIAL.

### The World's Columbian Exposition.

We observe with pleasure how rapidly the preparations for the World's Columbian Exposition are going forward, and the interest that seems to be increasing every month, as will appear from the following notices, which we think will prove of interest to some of the readers of the REGISTER:

Vermont will have a building at the Exposition without drawing on the State appropriation for the cost of its erection. One hundred substantial citizens have pledged themselves to pay $100 each.

Connecticut held an enthusiastic World's Fair Meeting at Hartford, on Washington's birthday, ex-Governor Waller presiding. A committee of sixteen, two from each county, were appointed to look after the State's representation at the Exposition. Sixteen lady managers were also chosen. Subscriptions being called for, $50,000 was pledged on the spot.

The Rhode Island building will combine the features of the "old stone mill" at Newport, which is of unknown origin, and which is alluded to in Longfellow's "Skeleton in Armor," and those of the "Arcade," a business building in Providence erected about sixty-five years ago.

Sir Henry Wood writes that applications for space are rapidly pouring in from influential firms in Great Britain. He is very enthusiastic over English prospects at the Exposition. Ceylon has sent through him a request for space upon which to build a tea house.

Commissioner McCormick writes from London that Mr. Armstrong will soon arrive in Chicago, his purpose being to present to the authorities of the Exposition a project to reproduce the Tower of London.

Denmark will spend about $5,500 in showing, as a leading feature of its exhibit, a Danish dairy, complete and in operation; the most approved methods and mechanical appliances are utilized in the dairies of that country.

The great dome of the Administration Building, which will be the most conspicuous architectural feature of the Exposition, and the four smaller domes, will be covered with aluminum bronze, a newly-discovered alloy, which is said to glisten brighter than gold. The American Sculptor MacMoines is in Paris working on the great fountain which will be placed in front of the Administration Building. The fountain will have thirty gigantic figures. Dan'l C. French, the New York sculptor, is also in Paris working on a colossal statue of the Republic for the World's Fair. It will show a female figure nearly seventy feet high.

The party under the direction of Chief Putnam, of the Ethnological Department of the Exposition, who had been making excavations of the mounds in Ohio, for several months, met with rare success near Chillicothe in making one of the richest finds of the century in the way of pre-historic remains. While at work on a mound 500 feet long, 200 feet wide and 28 high, the excavators found near the center of the mound, at a depth of 14 feet, the massive skeleton of a man encased in copper armor. The head was covered by an oval-shaped copper cap; the jaws had copper moldings; the arms were dressed in copper, while copper plates covered the chest and stomach, and on each side of the head on protruding sticks were wooden antlers ornamented with copper. The mouth was stuffed with genuine pearls of immense size, but much decayed. Around the neck was a necklace of bears' teeth, set with pearls. At the side of this skeleton was a female skeleton, the two supposed to be those of man and wife. It is estimated that the bodies were buried fully 600 years ago. The excavators believe they have at last found the king of the mound builders.

Russia, Turkey, Italy, Germany, Holland, Norway and Sweden are all busy planning and working for this great Exposition, and we hope from time to time to add a few more of the many items of interest concerning the work of other countries towards the success of this Great Fair. B.

